# Supplementary material for: Identification of photoperiod-induced specific miRNAs in the adrenal glands of Sunite sheep (Ovis aries)
Source: Front Vet Sci. 2022 Jul 22;9:888207. doi: 10.3389/fvets.2022.888207 (PMC9354845; doi:10.3389/fvets.2022.888207)
Supplement: Supplementary file 1 [file Data_Sheet_1.ZIP › Supplementary file/miRDeep2 file.zip!/miRDeep2 file/Novel_Structure/SPLP42A2/Novel_547.pdf]

Provisional ID	:	NC_019469.2_25317
Score total	:	12.1
Score for star read(s)	:	3.9
Score for read counts	:	4.7
Score for mfe	:	1.9
Score for randfold	:	1.6
Score for cons. seed	:	
Total read count	:	21
Mature read count	:	15
Loop read count	:	0
Star read count	:	6

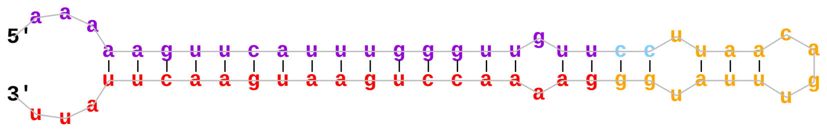

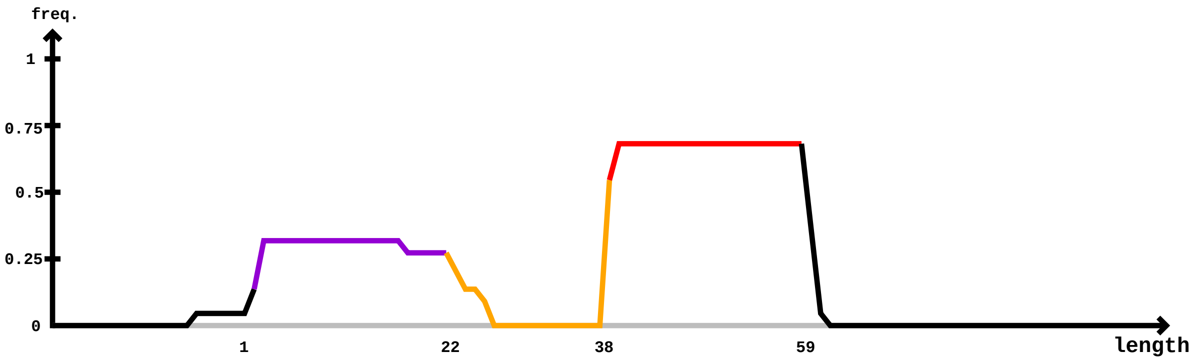

Star Mature

```
5'- uacuacuauuguguuggccaaaaaguucauuuggguuguuccuuaacaguuuauggggaaaaccugaaugaacuuauuggucaacccaauacgucagagaaauauagagugacag   -3'    obs
    uacuacuauuguguuggccaaaaaguucauuuggguuguuccuuaacaguuuauggggaaaaccugaaugaacuuauuggucaacccaauacgucagagaaauauagagugacag          exp
    ......(((((.(((((((((.(((((((((((((((.((((.(((....))).)))).))))))))))))))).))))))))).))))).((((.............))))..    reads     mm        sample
    .............uuggGcaaaaaguucauuuggg...............................................................................    1         1         seq
    ...................aaaaaguucauuuggguuguu..........................................................................    2         0         seq
    ....................aaaaguucauuuggguuguuG.........................................................................    1         1         seq
    ....................aaaaguucauuuggguuguuccA.......................................................................    1         1         seq
    ....................aaaaguucauuuggguuguuccuu......................................................................    2         0         seq
    ........................................................gaaaaccugaaugaacuuauu.....................................    11        0         seq
    ........................................................gaaaaccugaaugaacuuauug....................................    1         0         seq
    .........................................................aaaaccugaaugaacuuauug....................................    1         0         seq
    .........................................................aaaaccugaaugaacuuauuA....................................    1         1         seq
    .........................................................aaaaccugaaugaacuuauugg...................................    1         0         seq
```
